# The impact of artificial intelligence on behavioral intentions to use mobile banking in the post-COVID-19 era

**DOI:** 10.3389/frai.2025.1649392

**Published:** 2025-08-12

**Authors:** Johannes Schrank

**Affiliations:** ^1^International College, Khon Kaen University, Khon Kaen, Thailand; ^2^Center for Sustainable Innovation and Society, Khon Kaen University, Khon Kaen, Thailand

**Keywords:** mobile banking, artificial intelligence, FinTech, financial services, UTAUT

## Abstract

**Introduction:**

This quantitative research investigates the determinants of behavioral intentions to use mobile banking in the post-COVID-19 era. The study extends the Unified Theory of Acceptance and Use of Technology (UTAUT) framework by incorporating two key characteristics of AI, i.e. perceived intelligence and perceived anthropomorphism.

**Methods:**

It uses the UTAUT as a theoretical framework, and extends it by integrating core features of AI. Data has been collected from 412 respondents in Thailand, and structural equation modeling has been employed for the data analysis.

**Results:**

The findings reveal significant positive effects of performance expectancy, effort expectancy, social influence, facilitating conditions, trust, perceived privacy, perceived intelligence and anthropomorphism of AI on users’ behavioral intentions to use mobile banking. Price value, habits, and perceived security do not significantly influence behavioral intentions. The results highlight the transformative potential of AI technology in the mobile banking industry as consumers’ behaviors are greatly influenced by perceived intelligence and anthropomorphism.

**Discussion:**

The positive impact of perceived intelligence and anthropomorphism indicates that consumers value advanced, human-like interactions with AI. M-banking platforms may focus on developing AI systems that offer intuitive, intelligent, and emotionally engaging experiences. Financial institutions may invest in AI that can analyze user data to offer personalized financial advice, predict future needs, and automate routine tasks effectively.

## 1 Introduction

Nowadays, various technologies such as blockchain, artificial intelligence, and mobile platforms have increased convenience for consumers. In particular, the use of mobile phones to provide services such as financial transactions or mobile banking transactions have greatly enhanced convenience (Majumdar and Pujari, 2022). Furthermore, the COVID-19 pandemic has triggered a new normal lifestyle, which has accelerated the change in payment behavior of Thai consumers (Wongsansukcharoen, 2022). The COVID-19 pandemic caused social distancing measures and embraced the usage of mobile banking and the shift to online services. This influence may persist in the post-pandemic era (Zhu and Wang, 2022). Mobile banking adoption in Thailand is very high, 97% of Thai consumers use mobile banking applications at least once a week (Choomanee, 2024). This research can shed light on the financial transformation post-COVID-19 and help to understand what drives user intent in mobile banking. Additionally, the study focuses on artificial intelligence’s (AI) function in mobile banking as AI may create significant cost savings for financial institutions (Bredt, 2019). This study aims to examine the adoption of m-banking in the period following the COVID-19 pandemic by expanding upon the Unified Theory of Acceptance and Use of Technology (UTAUT) and by integrating artificial intelligence components. The COVID-19 pandemic significantly accelerated the adoption of digital technologies, including mobile banking (Chetioui et al., 2023). Social distancing measures, lockdowns, and the need for contactless transactions led to a surge in the use of mobile banking services. The pandemic forced individuals and businesses to rely heavily on digital services due to the need to minimize physical interactions. As a result, digital platforms, including mobile banking, became essential tools for conducting everyday transactions. This shift requires an updated understanding of the factors influencing technology adoption in this new context. The pandemic altered user expectations and behaviors. Users now prioritize convenience, safety, and reliability more than ever before. Understanding how these evolving expectations affect mobile banking adoption requires extending the existing theoretical framework of UTAUT. The incorporation of AI in mobile banking is a recent development that has transformative potential. AI capabilities, such as personalized recommendations, chatbots, and fraud detection, can significantly influence user perceptions and acceptance. By extending UTAUT to include AI characteristics, the study fills a gap in the existing literature and aims to capture the impact of these advanced technologies on user intentions. UTAUT traditionally focuses on performance expectancy, effort expectancy, social influence, and facilitating conditions (Baptista and Oliveira, 2015). However, the post-COVID-19 era presents new determinants, which are crucial for a comprehensive understanding of the mobile banking adoption. Understanding the factors that drive mobile banking adoption in this context can help providers and policymakers address current challenges and leverage opportunities. Extending UTAUT with relevant AI-related variables ensures that the framework remains applicable to current issues. While the traditional UTAUT model effectively explains technology adoption through constructs like performance expectancy, effort expectancy, and social influence, it does not fully capture the distinct attributes introduced by AI technologies. To address this gap, perceived intelligence and perceived anthropomorphism were incorporated as extensions to better reflect users’ interactions with AI-driven systems. These AI-specific factors differ from traditional UTAUT constructs by emphasizing emotional and cognitive user experiences rather than purely utilitarian or social aspects. Perceived anthropomorphism has been shown to increase social presence and rapport, enhancing user engagement and reducing anxiety toward automation (De Visser et al., 2016). These features can complement UTAUT factors by enhancing performance expectancy; for instance, an AI system perceived as intelligent and human-like may be seen as more effective and easier to use, thus strengthening adoption intentions. Studies highlight that anthropomorphic design elements increase user acceptance beyond what is explained by traditional technology acceptance models (Gnewuch et al., 2017). Therefore, integrating perceived intelligence and anthropomorphism enriches the explanatory power of UTAUT by capturing unique responses caused by AI systems.

This paper makes a valuable contribution to the theoretical advances of technology acceptance and adoption by expanding the UTAUT model. This analysis incorporates components from UTAUT, UTAUT2, and artificial intelligence. This study proposes that the core UTAUT factors (performance expectancy, effort expectancy, social influence, and facilitating conditions) positively influence behavioral intentions to use mobile banking. Building on UTAUT2, hedonic motivation, price value, and habits are also expected to have positive effects on behavioral intentions. Extending the model further, trust, perceived privacy, and perceived security are hypothesized to positively impact behavioral intentions. The artificial intelligence constructs of perceived intelligence and perceived anthropomorphism are predicted to enhance behavioral intentions, reflecting the growing role of AI in m-banking. Behavioral intentions are expected to positively influence the actual use behavior of mobile banking. Understanding the elements, which impact users’ behavioral intentions in the post-COVID-19 era is still lacking, despite the growing popularity of mobile banking and the increasing integration of artificial intelligence technologies in financial services. Although a large amount of research has been done on the adoption of m-banking, there is a lack of studies that have included AI-related aspects into their frameworks. Research that specifically examines how AI shapes users’ behavioral intentions toward mobile banking is needed, as the value of AI-driven features in improving user experiences and service efficiency grows. Thus, this study fills the gap by examining how behavioral intention is influenced by perceived intelligence and anthropomorphism. The COVID-19 pandemic has accelerated the adoption of mobile banking, with AI playing a crucial role in reshaping services. However, there remains a notable research gap regarding how AI’s capabilities are used to address the unique challenges posed by the pandemic and its aftermath. AI-driven solutions have enabled contactless interactions, enhanced operational efficiency, and provided personalized services, which are key factors in adapting to pandemic-induced shifts. In mobile banking, AI tools have automated processes, improved security, and personalized financial advice. Nevertheless, despite the growing use of AI, research has yet to fully address how these technologies have reshaped customer expectations in the post-COVID-19 period. This paper addresses how AI impacts the m-banking adoption in the post-COVID-19 era. A recent study has shown that AI can boost digital banking user satisfaction (Alnaser et al., 2023). However, there exists a research gap on the impact of AI on intentions to use m-banking. This paper fills the gap.

An empirical study has been conducted to address the following research purpose and questions:

- Research purpose: The study investigates the UTAUT’s determinants that impact users’ behavioral intentions to adopt m-banking in the post-COVID-19 era and extends the UTAUT model by incorporating two core features of AI, perceived intelligence and perceived anthropomorphism.
- Research question: Do the factors of the UTAUT framework and its extension (including artificial intelligence) positively affect the behavioral intention to use m-banking in the post-COVID-19 era?

## 2 Literature review

### 2.1 The impact of the COVID-19 pandemic on banking

The COVID-19 pandemic has greatly accelerated the shift toward mobile banking, as consumers sought convenient, safe, and accessible ways to manage their finances (Mu and Lee, 2022). With lockdowns and social distancing in place, mobile banking apps became essential for performing tasks like transferring funds, paying bills, and depositing checks without visiting branches. Because mobile payment platforms made it possible for users to avoid both direct and indirect connections, they were widely employed in daily life (Sleiman et al., 2023). This led to a significant rise in mobile banking adoption, particularly among those previously hesitant to use digital services (Eneizan et al., 2022). Banks responded by rapidly enhancing their mobile platforms, and by adding features like AI-driven financial tools, biometric authentication, and mobile check deposits. Consumers now expect seamless, real-time, and personalized experiences from their mobile banking apps, which make mobile banking a core part of the modern banking landscape (Manser Payne et al., 2021). Hence, the pandemic has strengthened mobile banking as the preferred method for many to manage their finances, which drives long-term changes in consumer behavior. During the COVID-19 outbreak, mobile payment usage has grown dramatically (Ali et al., 2024). The fear of virus transmission through cash transactions significantly drove users toward mobile banking as a safer alternative (Sebayang et al., 2024). The convenience of conducting transactions from home without physical interaction was a significant motivator for many users (Zeya, 2022). Further, the COVID-19-induced adoption of AI in mobile banking has led to an increased customer acquisition and retention (Shankar et al., 2023) as AI can enhance the satisfaction of digital banking users (Alnaser et al., 2023). Therefore, the banking sector may concentrate on innovative AI technologies to improve customer services (Noreen et al., 2023). In Thailand, the COVID-19 pandemic has expedited the shift in digital banking toward mobile banking (Silanoi et al., 2023).

### 2.2 Unified theory of acceptance and use of technology (UTAUT)

The Unified Theory of Acceptance and Use of Technology has served as a foundational framework for understanding technology adoption behavior, including mobile banking. According to UTAUT, performance expectancy (PE), effort expectancy (EE), social influence (SI), and facilitating conditions (FC) are primary determinants of users’ behavioral intentions to use technology (Venkatesh et al., 2003). UTAUT has been widely used in information systems and other fields (Venkatesh et al., 2016). The theory was created by reviewing and combining the concepts of eight models, such as technology acceptance model and theory of planned behavior, that were used in previous studies to explain the behavior of users of information systems. After being validated by Venkatesh et al. (2003), UTAUT was found to explain approximately 50% of the variance in actual usage and 70% of the variance in behavioral intention to use. Numerous studies have validated the applicability of UTAUT in the context of mobile banking and highlighted the significance of these factors in shaping users’ attitudes and intentions toward mobile banking adoption (Lai and Li, 2005; Zhou, 2013). UTAUT is one of the main conceptual frameworks adopted by researchers to explain consumers’ use or intention to use m-banking (Souiden et al., 2021). UTAUT provides a comprehensive framework that encompasses multiple dimensions which influence technology adoption. It offers a wide view of user behavior. Moreover, UTAUT is relevant to the study of modern technology use, including mobile banking, as it addresses key factors that influence user acceptance. UTAUT has strong empirical support as it has been validated across various contexts and technologies. This reliability makes it a trusted model for studying mobile banking usage (Jadil et al., 2021). Further, UTAUT’s flexibility allows for the inclusion of additional variables to better capture the mobile banking adoption, particularly in the post-COVID-19 context. The UTAUT not only identifies key constructs that influence mobile banking adoption, but also emphasizes the underlying mechanisms and contextual factors that shape user behavior and decision-making processes. It can be applied to accurately predict customer behavioral intentions toward mobile banking (Farzin et al., 2021).

Performance expectancy, or the belief that mobile banking will enhance task efficiency, has a strong positive impact on users’ behavioral intentions to adopt and use the service. When users perceive that mobile banking improves convenience, saves time, and provides better control over their finances, they are more likely to adopt it. Chan et al. (2012) studied the acceptance and spread of the use of collaboration technology via electronic media among small and medium-sized businesses. The outcomes show that the factors of performance expectancy influence the adoption of electronic collaboration technology. Naruetharadhol et al. (2023) found that performance expectancy has a positive impact on behavioral intentions to use an e-commerce platform.

Extending the UTAUT theoretical framework to explain individual technology adoption and use behavior, it was found that effort expectancy factors significantly affect usage (Venkatesh et al., 2012). Effort expectancy, which refers to the ease of use associated with mobile banking, positively influences users’ behavioral intentions to adopt the service. When users perceive that mobile banking is simple, intuitive, and easy to navigate, they are more likely to use it. Research comparing technology adoption has found that effort expectancy factors directly influence technology adoption and usage behavior. When users feel that technology does not require much effort, users’ expectations for work efficiency will be high (Siswanto et al., 2018). In addition, research on the background of m-banking has confirmed that effort expectancy positively impacts behavioral intentions to use m-banking services (Bankole et al., 2011).

Social influence involves the conformity to reference groups, social factors, and social image. Alalwan et al. (2016) define in the context of mobile financial services, social influence refers to how customers’ willingness to use mobile banking is impacted by their social surroundings. When most users lack information and experience about various techniques, they must rely on the opinions of close environments such as family, friends or superiors. This affects the perception of various behaviors related to certain activities (Kalinic and Marinkovi, 2016). Similarly, there is research indicating that social influence in the form of feedback from others can affect the aim to use m-banking (Kosim and Legowo, 2021).

Facilitating conditions refer to the degree of people who believe that technical and organizational readiness exists to justify the utilization of a system. The factor facilitating conditions is one of the factors that affect users’ beliefs about available facilities and affect their intention to use technology. A study by Farzin et al. (2021) proved that facilitating conditions are a factor in the use and acceptance of technology. A variety of skills are required for instance, the installation of banking applications, acquaintance about mobile operations services, and safety features (Purwanto and Loisa, 2020).

*H1:* Performance expectancy has a positive effect on behavioral intentions.

*H2:* Effort expectancy has a positive effect on behavioral intentions.

*H3:* Social influence has a positive effect on behavioral intentions.

*H4:* Facilitating conditions have a positive effect on behavioral intentions.

### 2.3 UTAUT2

Building upon UTAUT, researchers have extended the framework to incorporate additional constructs. UTAUT2 introduces hedonic motivation (HM), price value (PV), and habits (HT) as supplementary factors, which influence users’ behavioral intentions (Venkatesh et al., 2012). UTAUT2 aims to better understand consumer acceptance and use of technology, and refines the original model to fit the context of consumer technology adoption. Both UTAUT and UTAUT2 have been widely applied to study the adoption of various technologies, and have been validated in different cultural contexts, which demonstrates the models’ robustness and adaptability across diverse populations and settings. The models provide a robust theoretical framework for studying technology acceptance. They guide researchers in exploring new determinants, such as AI factors, and extending the models to new technologies. Studies exploring UTAUT2 in the background of m-banking have highlighted the importance of hedonic motives, perceived value, and habitual usage patterns in driving users’ adoption and usage behavior (Zhou, 2012; Wang et al., 2006). Hedonic motivation, which refers to the enjoyment or pleasure derived from using technology, has a positive effect on behavioral intentions to use mobile banking. When users find mobile banking to be engaging or enjoyable, whether through a pleasing interface, features, or interactive elements, they are more inclined to adopt the service. This sense of enjoyment enhances the overall user experience.

The notion of price value is central to users’ assessments of the advantages they derive from adopting and using a technology like m-banking. Price value has emerged as an essential determinant in individuals’ decisions regarding mobile banking adoption (Zeya, 2022). Users are assessing whether the benefits of mobile banking, such as its simplicity and security when conducting contactless transactions, outweigh any costs or fees. The perceived price value of a product has become a crucial determinant in the adoption decisions of users.

Habits, defined as automatic behaviors developed through repeated use, positively influence behavioral intentions to use mobile banking. When users frequently engage with mobile banking services, it becomes a routine part of their daily activities, which reduces the effort needed to perform financial tasks. Due to a number of important considerations, the role of habit in using m-banking has increased dramatically in the post-COVID-19 period. Significant changes in user behavior were brought forth by the COVID-19 epidemic, including a quick switch to online banking and remote financial transactions. As users adapted to these new routines amidst lockdowns and social distancing measures, the habitual use of mobile banking apps became more prevalent and essential (Sunarjo et al., 2021).

*H5:* Hedonic motivation has a positive effect on behavioral intentions.

*H6:* Price value has a positive effect on behavioral intentions.

*H7:* Habits have a positive effect on behavioral intentions.

### 2.4 The extension

Trust (T), perceived privacy (PP), and perceived security (PS) are critical considerations that affect users’ willingness to engage with m-banking platforms. The theoretical foundation supporting the variables Trust, Perceived Privacy, and Perceived Security is rooted in the established framework of the extended UTAUT. Existing studies extend the UTAUT model by incorporating these variables (Hanif and Lallie, 2021; Widyanto et al., 2022). Trust plays a crucial role in positively influencing behavioral intentions to use mobile banking. When users perceive mobile banking platforms as secure, reliable, and capable of protecting their personal and financial information, their confidence in the service increases. This trust, in turn, encourages them to adopt and continue using mobile banking for transactions and other financial activities. Research has identified trust as a key determinant of users’ behavioral intentions, which emphasizes the importance of perceived reliability, credibility, and integrity of the service provider (Kim and Prabhakar, 2004). Addressing users’ trust, privacy, and security is essential for building confidence and promoting adoption of m-banking services. Trust is essential in the adoption and continued use of m-banking (Alalwan et al., 2017). Users must trust that their financial information and transactions are secure, especially when conducting banking activities through mobile apps. In the post-COVID-19 era, where digital banking has gained even more prominence, trust becomes crucial. More people chose to use mobile banking after the COVID-19 outbreak as online transactions were the only option during the lockdown measures (Naeem and Ozuem, 2021). Trust continues to be a critical component in the uptake of m-banking.

The concept of perceived privacy in mobile banking has taken importance as digital financial interactions have become the norm (Smith et al., 2012). Several critical factors contribute to these perceived privacy concerns. Data security, as highlighted by Xu et al. (2012), stands out as a foremost worry among users who fear data breaches and unauthorized access to their sensitive information. The transparency of privacy policies and data handling practices, as emphasized by Foroughi et al. (2019) significantly influences users’ trust and perception of privacy. Empirical research has demonstrated the positive impact of addressing these concerns on user trust and overall acceptance of mobile banking services (Chakiso, 2019). Perceived privacy involves users’ beliefs about how their personal and financial data are collected, used, and shared. In the digital age, and particularly post-COVID-19, concerns about data privacy have become more pronounced due to increased digital interactions and data breaches. With more personal data being shared online, users are increasingly sensitive about how their information is handled. Users who trust that their data is handled responsibly are more likely to engage with mobile banking services. Concerns about privacy can act as a barrier to adoption.

In the post-COVID-19 era, perceived security plays a central role in users’ decision-making processes. Customers are more inclined to trust m-banking systems when they perceive them as secure and resistant to threats such as fraud and data breaches (Alalwan et al., 2017). Similarly, trust in m-banking providers is closely tied to the perception of robust security measures (Du and Agami, 2017). User education plays a vital role, with a focus on providing clear information about security features and practices (Laukkanen, 2016). Transparency in disclosing privacy policies and data handling practices, as highlighted by Du and Agami (2017), fosters trust and eases concerns. Perceived security refers to users’ confidence that their financial transactions and personal information are safe from threats such as fraud, hacking, and unauthorized access. This aspect has always been crucial, but its importance has been increased post-COVID-19 due to the significant increase in online transactions and the parallel rise in cyber threats. The pandemic led to a surge in m-banking transactions, which makes security a concern for users who previously relied on physical banking. High perceived security builds trust, which is essential for retaining existing users and attracting new ones. Consequently, perceived risk and technology anxiety have a negative effect on the intention to use mobile payment (Schrank, 2025).

*H8:* Trust has a positive effect on behavioral intentions.

*H9:* Perceived privacy has a positive effect on behavioral intentions.

*H10:* Perceived security has a positive effect on behavioral intentions.

### 2.5 Artificial intelligence

Modern developments in AI have significantly enhanced mobile banking services, which improve both customer satisfaction and service efficiency (Alnaser et al., 2023). AI can influence m-banking user acceptance as it enables mobile banking applications to deliver personalized services based on user behavior, preferences, and transaction history. Machine learning algorithms can analyze data to offer customized recommendations, financial insights, and predictive analytics (Portugal et al., 2018). This personalization enhances user satisfaction and engagement, and thereby increases acceptance and adoption of mobile banking services. AI-powered chatbots and virtual assistants provide real-time customer support, which can answer queries, resolve issues, and assist with transactions. These AI interfaces simulate human-like interactions, and offer 24/7 availability and quick response times for mobile banking customers. Such capabilities improve user trust and satisfaction by ensuring reliable and efficient customer service experiences and therefore, influence user acceptance. AI enhances m-banking security through advanced fraud detection algorithms that analyze transaction patterns and detect anomalies in real-time. AI can also strengthen authentication methods with biometric recognition, which make transactions more secure and reduce the risk of fraud. Improved security measures build user confidence in mobile banking platforms, which is crucial for adoption and acceptance. Moreover, AI optimizes processes in mobile banking, such as data processing, compliance monitoring, and risk management. Automation of routine tasks reduces operational costs and enhances efficiency (Acemoglu and Restrepo, 2018). This allows financial institutions to allocate resources more strategically. These operational efficiencies can lead to lower service fees and improved service delivery, which may positively influence user acceptance. The Theory of Computation (ToC) provides essential insights into algorithmic processes, including automata theory, computability, and complexity theory, which are crucial for modeling AI systems (Chaurasia et al., 2024). Studies in service automation, and social robotics (Gursoy et al., 2019) have demonstrated that perceived intelligence and anthropomorphism can enhance perceived usefulness, trust, and behavioral intention.

A study examining the social support theory have identified perceived intelligence and anthropomorphism as factors influencing consumers’ perception of social support, which sequentially impact users’ mobile banking usage (Lin and Lee, 2023). Development of artificial intelligence is opening new doors in the lives of many people (Sanayei et al., 2023) and is changing the consumer–brand interactive relations (Lim et al., 2022). The application of AI in financial services includes features such as chatbots and virtual assistants, fraud detection, personalized banking, process automation, etc. (Riikkinen et al., 2018). Perceived intelligence and anthropomorphism can build attitude and the intention to use chatbot-based services (Balakrishnan et al., 2022). Further, perceived intelligence and anthropomorphism are significant determinants of the adoption of personal intelligent agents (Moussawi et al., 2021). Attitude toward AI significantly impacts the intention to use AI in banking services (Rahman et al., 2023). When AI is included into a digital self-service technology channel, service delivery and the customer’s participation in value co-creation change (Manser Payne et al., 2021). Perceived intelligence pertains to users’ perceptions of AI’s capability to perform tasks effectively, such as providing accurate financial advice, personalizing user experiences, and resolving issues efficiently. AI’s role in mobile banking has grown significantly post-COVID-19. Intelligent AI systems can greatly enhance the user experience by offering personalized services and efficient problem-solving. Hence, perceived intelligence is of great significance to the m-banking usage phenomenon.

Perceived anthropomorphism in AI relates to the degree users perceive or attribute human-like qualities, behaviors, or characteristics to the AI systems they are interacting with. The interactions feel less like a robot and more like a human connection, which can lead to user trust and a greater willingness to interact with AI. Thus, users feel more like they are interacting with living beings than with machines (Adam et al., 2021). The anthropomorphic profile design of chatbots can enhance social presence communication’s positive effects (Tsai et al., 2021). However, when sending information or advice, users are concerned about their privacy, permissions, and the potential for manipulation or deception by AI (Hermann, 2022). Moreover, human intervention in AI-driven banking is important and can be accomplished by personalized service experience features (Sheth et al., 2022). Nevertheless, perceived anthropomorphism increases consumers’ willingness to adopt mobile banking apps based on the stimulus-organism-response theory (Lee and Chen, 2022). Perceived anthropomorphism involves attributing human-like qualities to AI, which makes interactions with mobile banking systems more natural and engaging. This aspect has gained importance as AI becomes more integrated into user interactions. It is significant in the post-COVID-19 era as anthropomorphic AI can enhance user engagement by making interactions more relatable and less mechanical and anthropomorphic features can make technology more accessible to a wider range of users, including those who may be less open to new technologies.

*H11:* Perceived intelligence has a positive effect on behavioral intentions.

*H12:* Perceived anthropomorphism has a positive effect on behavioral intentions.

### 2.6 Use behavior

Use behavior is affected by the behavioral intention toward m-banking, which is influenced by a number of variables. Users’ behavioral intentions to utilize mobile banking services positively influence their actual use behavior. It builds upon the theory of planned behavior (Ajzen, 1991), which suggests that individuals are more likely to engage in a behavior if they have a strong intention to do so. Numerous studies have demonstrated a significant positive relationship between users’ intentions to use m-banking and their subsequent adoption and usage behavior (Luarn and Lin, 2005).

*H13:* Behavioral intentions have a positive effect on use behavior of m-banking.

In the aftermath of the COVID-19 pandemic, consumer behavior toward digital financial services has undergone a significant transformation, driven by the accelerated adoption of contactless and remote banking solutions. The empirical model in this study reflects this shift by examining the factors that influence behavioral intentions to use mobile banking in the post-COVID-19 era. Building on the UTAUT and UTAUT2 frameworks, the model incorporates pandemic-relevant extensions such as trust, perceived privacy, and perceived security, which have become critical as users increasingly demand reliable and secure digital experiences. Moreover, the model integrates perceived artificial intelligence and perceived anthropomorphism to capture the growing reliance on AI-driven interactions during and after the pandemic, as financial institutions adopted intelligent systems to maintain service continuity. By linking these variables to behavioral intention and actual usage, the model provides a comprehensive view for understanding mobile banking adoption in a period marked by heightened digital dependency, risk awareness, and changing expectations for personalized and intelligent financial services.

**Figure 1:**
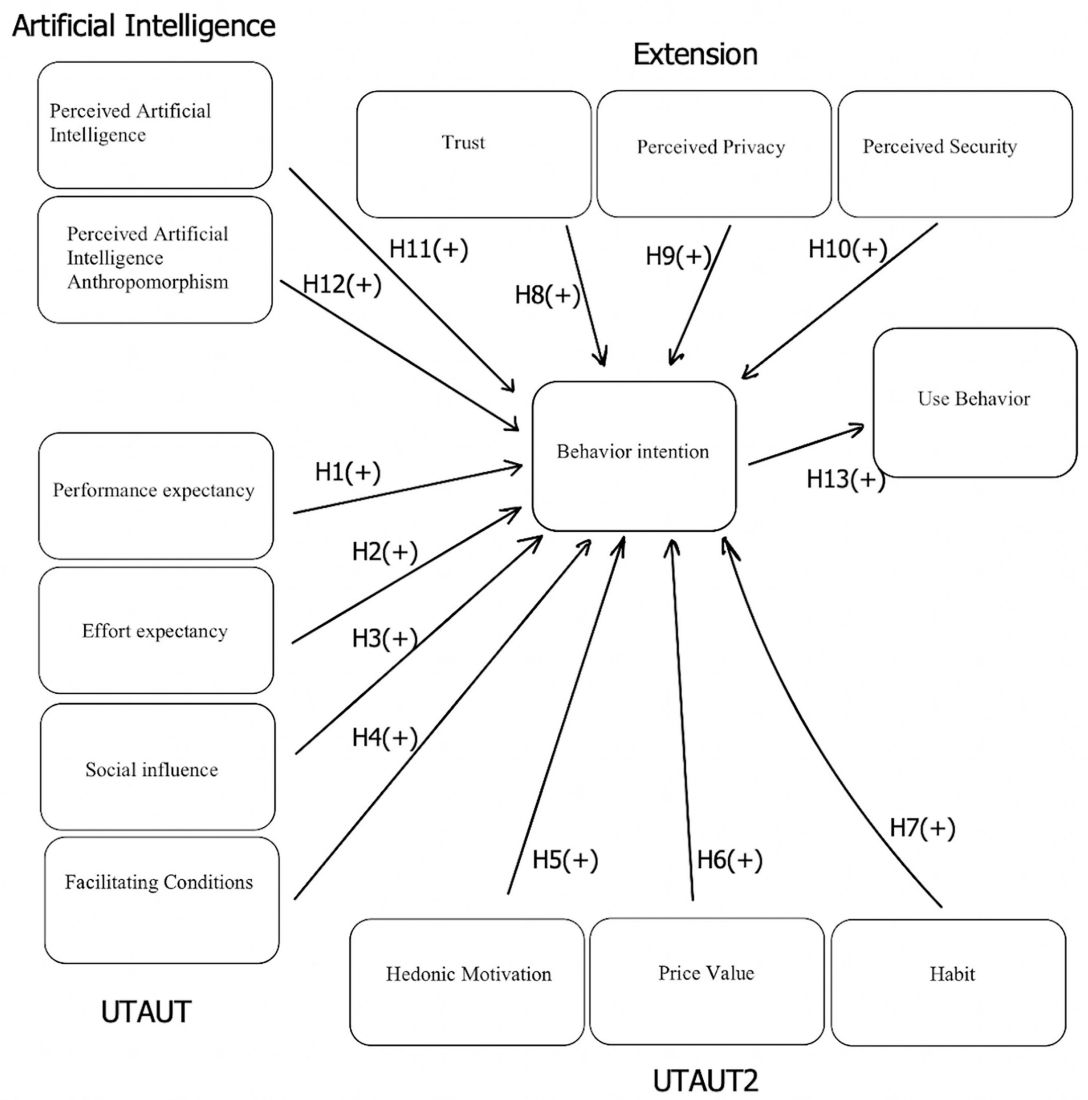
Research model.

The hypotheses have been incorporated in the research framework as shown in Figure 1.

## 3 Research methodology

### 3.1 Data collection

This study uses online questionnaires as a tool to collect data. The chosen sampling method is random sampling. It ensures that the findings are robust, reliable, and generalizable to the broader population. Random sampling reduces selection bias as it guarantees that every member of the population has an equal chance of being included in the sample. This leads to more accurate and reliable results. Since each member of the population has an equal chance of being selected, the sample is more likely to be representative of the entire population. Participants for the questionnaire were randomly selected from a pool of volunteers who expressed willingness to participate. The data was gathered from 430 respondents in Thailand. 412 replies remained useful after errors, outliers, and irrelevant data were eliminated. As a result, 4.2% of the sample is invalid. The recruitment period for this study started on 10th January 2024 and ended on 30th April 2024. Data collection was conducted in the post-COVID-19 era, a time when digital services, including mobile banking, became increasingly essential. The pandemic accelerated the shift toward contactless financial solutions, making users more familiar with and dependent on mobile banking platforms. This context justified the use of online surveys and a focus on AI-driven features, as consumers had greater exposure to digital tools and were better positioned to assess human-like interactions in mobile banking. For the data collection, Google Forms surveys were created and distributed over social media networks such as Facebook, Instagram, and Line, among others. To enhance validity, the instrument was pretested with a pilot sample of 30 respondents, after which ambiguous wording was refined. Content validity was ensured by adapting established constructs from the UTAUT and AI literature and consulting with academic experts in information systems and digital banking. Reliability and construct validity were assessed using Cronbach’s alpha, exploratory factor analysis, and confirmatory factor analysis. To verify the authenticity of responses and reduce careless answering, attention-check questions were embedded. The final dataset included only complete responses that passed consistency and attention filters, ensuring a high-quality dataset suitable for structural equation modeling. The questionnaire was translated from English to Thai for the convenience of Thai respondents. The population in this study refers to the collection of individuals who have ever used mobile banking. All respondents were asked to answer the questionnaire truthfully and completely. Furthermore, all information is kept strictly confidential and is not disclosed to the public for any reason. It has been confirmed that the sample is representative. The features of the sample can fairly represent the population. Furthermore, a more representative sample was produced by the large sample size of 412 respondents. The age distribution is slightly dominated by young people aged 18–29 as the study targets consumers that have experience in using m-banking. This age group tends to use mobile devices more frequently for various activities, including financial transactions, communication, and social networking. Younger people are typically more comfortable with technology and tend to adopt new digital platforms and innovations more quickly than older age groups. They are often early adopters of technologies like mobile banking, AI-powered services, and other digital solutions. Further, the researcher has chosen to use five job divisions. These broad, generalized job divisions can simplify the classification of respondents. These five categories cover major economic statuses, and capture a wide range of individuals based on their employment type, rather than focusing on more specific or niche occupations. These job divisions are common in Thailand (Srisathan and Naruetharadhol, 2022). Table 1 displays the demographic information of the sample.

**Table 1:** Demographics of the respondents.

Demographics		Number	Percentage
Gender	Male	195	47.3%
	Female	217	52.7%
Age	18–29 years	212	51.5%
	30–39 years	115	27.9%
	40–49 years	52	12.6%
	50–59 years	18	4.4%
	Above 60	15	3.6%
Education	High school or below	84	20.4%
	Bachelor’s degree	272	66.0%
	Master’s degree	44	10.7%
	Doctor of Philosophy	12	2.9%
Employment	Student	137	33.3%
	Government official	96	23.3%
	Company employee	77	18.7%
	Self-employed	73	17.7%
	Unemployed	29	7.0%

### 3.2 Questionnaires

I executed a pilot study by collecting data from 30 participants in Thailand to ensure that the questions do not contain errors. A test was performed to confirm the reliability of the questionnaire using Cronbach’s alpha correlation coefficient. The results are consistent with the theory that the reliability must be greater than 0.70 (Cortina, 1993). Questionnaires served as the mean of data collection. A robust questionnaire was created by defining clear objectives, conducting a literature review, and drafting questions as described in the first and second section. A thorough literature study formed the basis for the questionnaire items and provided the research with a solid framework. This review was important in guiding the construction of the research questions, aligning them with the established research model, and ensuring their relevance to the study’s objectives. To ensure validity, experts reviewed the questionnaire draft for relevance, clarity, and comprehensiveness, and provided feedback for revisions. After adjustments based on the recommendations had been made, pre-testing followed. The questionnaire was administered to a small, representative sample of 30 people to identify and address any ambiguities or issues. Feedback from pre-testing helped to refine the questions further in order to ensure they are clear and effective in capturing the intended information without any errors and inaccuracies. The final questionnaire was then formatted for easy reading and completion. It adheres to ethical standards such as informed consent and respondent confidentiality, and was distributed to potential respondents. Khon Kaen University Ethics Committee for Human Research, Khon Kaen University, Khon Kaen, Thailand, has made an agreement that this study has met the criteria of the Exemption Determination Regulations on 8 December 2023 (HE663371). Furthermore, written informed consent was obtained from all subjects involved in the study. The questions in the questionnaire are divided into two parts as follows: Part 1: Questionnaire on general information of respondents (gender, age, education, employment), Part 2: Questionnaire on factors influencing behavioral intentions to use mobile banking (see Appendix 1). The measurement items used for all constructs (including the AI variables) are shown in Appendix 1. It also lists the references. The questionnaire uses the Likert rating scale to evaluate the results with a 7-rating scale, ranging from 1 = strongly disagree to 7 = strongly agree. This article uses SPSS and AMOS to analyze the data.

### 3.3 Structural equation modeling

The structural equation modeling (SEM) method was employed for the data analysis in the study. SEM includes a wide range of statistical methods, including causal modeling with latent variables, confirmatory factor analysis and path analysis. The model’s estimation was assessed using the SEM in two stages, i.e., reliability and validity of each indicator’s variable, and the overall reliability of the structure (Anderson and Gerbing, 1988).

## 4 Results

### 4.1 Exploratory factor analysis

Although the study is based on established constructs from the UTAUT framework and related literature, exploratory factor analysis (EFA) is conducted as a preliminary step to assess the factor structure. In particular, the inclusion of newer constructs, such as perceived intelligence and perceived anthropomorphism, warranted an empirical examination of item behavior and dimensional validity. EFA is used to confirm whether items loaded appropriately on their intended factors and to ensure no unexpected cross-loadings emerged. No items are removed, and the original factor structure is retained, confirming the suitability of the measurement model prior to confirmatory factor analysis (CFA). The identity matrix has been checked using the Bartlett’s sphericity test and the Kaiser–Mayer–Olkin (KMO) test (Taherdoost et al., 2022). The KMO result is 0.911. Since the KMO value is greater than 0.50, the sample adequacy conditions are satisfied. The *p* value of the Bartlett’s test of sphericity is <0.001. The statistical significance of the Bartlett’s test of sphericity indicates that the correlation matrix differs from an identity matrix as intended. The maximum likelihood approach with varimax rotation is used in the exploratory factor analysis to examine the correlation and factor structure among the items. The outcomes of the rotated factor matrix show that all items are loading on their own factors. The results of the exploratory factor analysis show that the factors have a satisfactory level of validity.

### 4.2 Confirmatory factor analysis

Based on the provided fit indices for the measurement model, the model meets the recommended thresholds, which indicates an acceptable fit to the data. The model fit results (CMIN/DF = 2.167, NFI = 0.913, CFI = 0.926, GFI = 0.921, TLI = 0.916, AGFI = 0.841, RMR = 0.042, RMSEA = 0.054) passed the thresholds (Kline, 2005). Based on these fit indices, the measurement model provides a satisfactory representation of the relations between the observed variables and the underlying constructs in the data.

As shown in Table 2, all structures have a Cronbach’s Alpha larger than the suggested value of 0.70 (Saunders et al., 2003). Furthermore, the AVE values of the entire structure are larger than the recommended value level of 0.50 (Fornell and Larcker, 1981). The CR values of the structure are greater than the suggested value level of 0.70. Additionally, the loadings exceed the recommended value of 0.70, which indicates a high-level relation between the variables (Tabachnick and Fidell, 2013). Hence, the results show that all structures within the model exhibit robust reliability and convergent validity. These findings support the measurement model’s capacity to evaluate the latent constructs with accuracy and support the validity of the relationships hypothesized in the research model.

**Table 2:** Convergent validity.

Variable	Item	Loadings	AVE	CR	Cronbach’s Alpha
Performance expectancy	4	0.820–0.836	0.686	0.897	0.896
Effort expectancy	6	0.794–0.828	0.662	0.922	0.920
Social influence	3	0.774–0.815	0.639	0.841	0.840
Facilitating conditions	4	0.796–0.841	0.671	0.891	0.891
Hedonic motivation	3	0.786–0.838	0.653	0.849	0.848
Price value	3	0.809–0.837	0.672	0.860	0.860
Habits	3	0.765–0.810	0.618	0.829	0.826
Trust	3	0.850–0.864	0.736	0.893	0.891
Perceived privacy	5	0.833–0.871	0.738	0.934	0.934
Perceived security	3	0.831–0.867	0.725	0.888	0.884
Perceived intelligence	3	0.743–0.763	0.568	0.797	0.797
Perceived anthropomorphism	3	0.702–0.716	0.501	0.751	0.751
Behavioral intention	3	0.732–0.741	0.541	0.780	0.777
Use behavior	3	0.808–0.824	0.667	0.857	0.856

The discriminant validity (DV) has been verified using the Heterotrait-Monotrait (HTMT) ratio. I confirmed DV by the HTMT ratio as shown in Table 3. The HTMT ratio is the degree to which a construct is empirically distinct from other constructs in the measurement model (Hair et al., 2019). As per the recommendation of Henseler et al. (2015), valid values must be below 0.85. The Fornell-Larcker criterion is a commonly used method for evaluating discriminant validity (Fornell and Larcker, 1981). The Fornell-Larcker criterion, however, lacks specificity when combined with consistent estimates (Voorhees et al., 2016) and sensitivity when combined with varianced-based structural equation modeling results (Rönkkö and Evermann, 2013). Thus, this study utilizes the HTMT method.

**Table 3:** Heterotrait-Monotrait ratios.

Construct	PE	EE	SI	FC	HM	PV	HB	T	PP	PS	PI	PA	BI	UB
PE	–													
EE	0.796	–												
SI	0.765	0.749	–											
FC	0.704	0.817	0.818	–										
HM	0.654	0.779	0.759	0.726	–									
PV	0.720	0.778	0.694	0.737	0.777	–								
HB	0.676	0.748	0.734	0.781	0.795	0.768	–							
T	0.584	0.652	0.588	0.634	0.792	0.555	0.712	–						
PP	0.666	0.693	0.622	0.682	0.787	0.557	0.668	0.819	–					
PS	0.586	0.750	0.703	0.706	0.797	0.598	0.679	0.821	0.820	–				
PI	0.764	0.789	0.814	0.818	0.709	0.766	0.772	0.640	0.660	0.756	–			
PA	0.729	0.731	0.766	0.761	0.767	0.727	0.752	0.682	0.662	0.707	0.815	–		
BI	0.740	0.733	0.668	0.748	0.786	0.785	0.810	0.600	0.586	0.636	0.813	0.799	–	
UB	0.659	0.699	0.629	0.685	0.649	0.672	0.672	0.481	0.548	0.533	0.761	0.699	0.823	–

PE, Performance Expectancy; EE, Effort Expectancy; SI, Social Influence; FC, Facilitating Conditions; HM, Hedonic Motivation; PV, Price Value; HB, Habits; T, Trust; PP, Perceived Privacy; PS, Perceived Security; PI, Perceived Intelligence; PA, Perceived Anthropomorphism; BI, Behavioral Intention; UB, Use Behavior.

In this study, common method variance (CMV) has been examined. I used the single-factor test of Harman following Podsakoff et al. (2012). The findings confirmed the absence of CMV by revealing a total variance of 45.588 percent, which is less than the 50 percent threshold. To verify the absence of multicollinearity, the article used Pearson’s product moment correlations, which are below the threshold of 0.80 (Franke, 2010).

### 4.3 Results of the structural model

Based on the fit indices provided for the structural model, the model meets the recommended thresholds, which indicates an acceptable fit to the data. The model fit results (CMIN/DF = 2.458, NFI = 0.922, CFI = 0.901, GFI = 0.912, TLI = 0.932, AGFI = 0.815, RMR = 0.063, RMSEA = 0.060) passed the thresholds (Kline, 2005). Therefore, the model can be considered valid and reliable for analyzing the hypothesized relationships and drawing meaningful conclusions in the research context.

The squared multiple correlation value (*R*^2^) of behavioral intention is 0.813 and of use behavior 0.518, respectively. 81.3 percent of the variance in behavioral intention is explained by the independent variables. 51.8 percent of the variance in use behavior is explained by behavioral intention. Therefore, there is a good fit of the model as latent constructs explain the observed variables well. The values indicate a strong explanatory power and a strong relationship between predictors and the endogenous variable.

**Figure 2:**
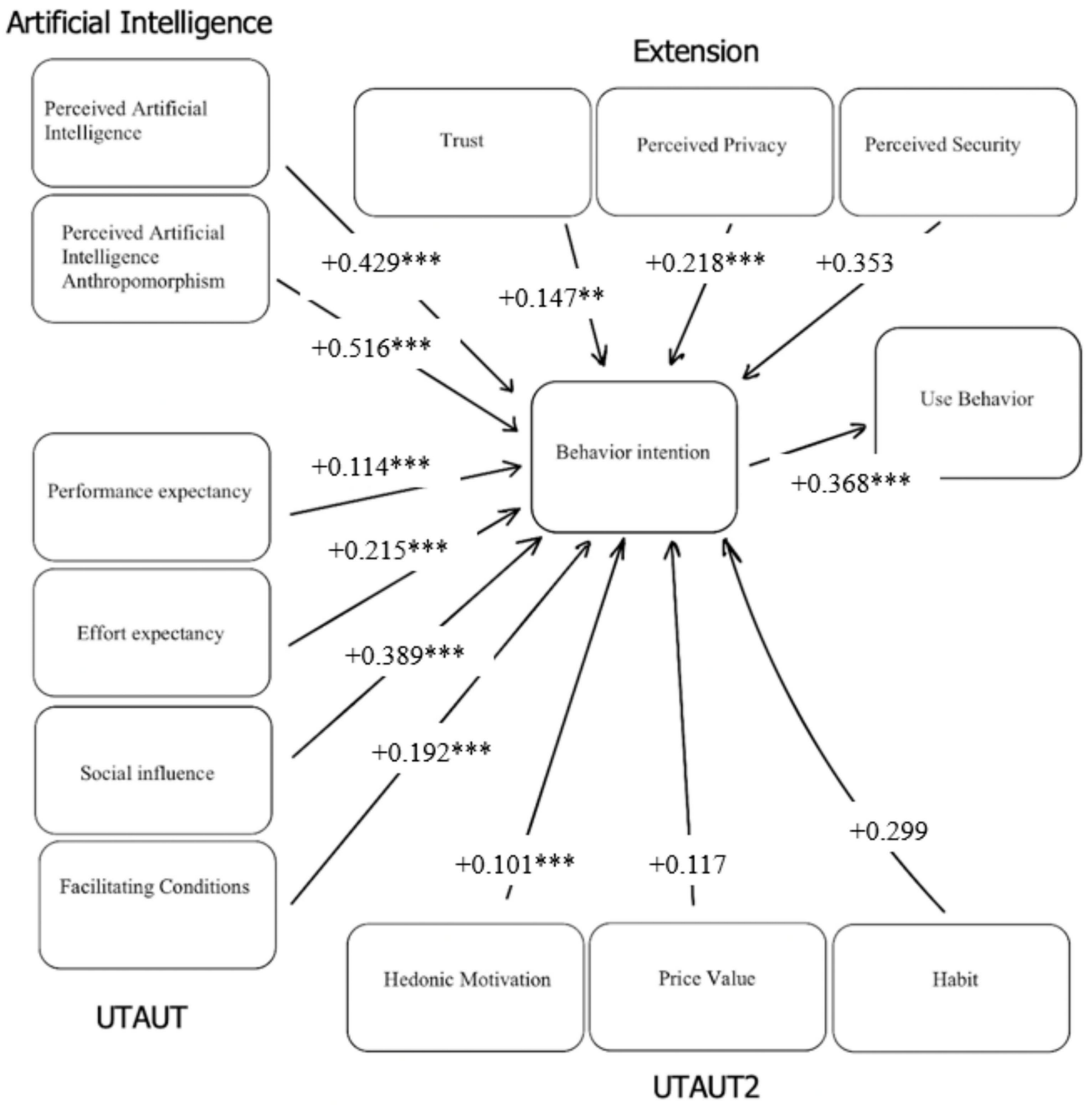
Research model with the hypothesis test results. ***/**/* specify significance at 99 percent, 95 percent, and 90 percent confidence levels. The figure illustrates the path coefficients and significance levels.

Table 4 summarizes the results of the hypothesis tests. The results reveal significant positive effects of several key determinants on users’ intentions to engage with mobile banking platforms. Performance expectancy demonstrates a significant positive influence on behavioral intentions to use m-banking (H1: *β* = 0.114, *p* < 0.001). Similarly, effort expectancy significantly influences users’ behavioral intentions (H2: *β* = 0.215, *p* < 0.001). Social influence also emerges as a significant driver of behavioral intentions toward mobile banking (H3: *β* = 0.389, *p* < 0.001). Additionally, facilitating conditions significantly contribute to shaping users’ intentions (H4: *β* = 0.192, *p* < 0.001). Furthermore, the analysis highlights the importance of psychological factors in influencing users’ intentions. Hedonic motivation demonstrates a significant positive effect on behavioral intentions (H5: *β* = 0.101, *p* = 0.006). Trust in mobile banking platforms and perceived privacy also emerge as significant predictors of behavioral intentions (H8: *β* = 0.147, *p* = 0.022; H9: *β* = 0.218, *p* = 0.003). Moreover, perceived intelligence and anthropomorphism of artificial intelligence integrated into mobile banking platforms significantly influence users’ behavioral intentions (H11: *β* = 0.429, *p* < 0.001; H12: *β* = 0.516, *p* < 0.001). Additionally, behavioral intentions themselves have a significant positive impact on the actual use behavior of m-banking (H13: *β* = 0.368, *p* < 0.001). Factors such as price value and habits do not demonstrate significant effects on behavioral intentions (H6: *β* = 0.117, *p* > 0.10; H7: *β* = 0.299, *p* > 0.10). Similarly, the perceived security of m-banking platforms does not significantly influence behavioral intentions (H10: *β* = 0.353, *p* > 0.10). Figure 2 illustrates the results of the hypothesis tests.

**Table 4:** Results of the hypothesis tests.

Hypothesis	Standardized estimate	*T* value	*p* value	Result
H1: Performance Expectancy has a positive impact on Behavioral Intentions.	0.114	10.412	0.000***	Supported
H2: Effort Expectancy has a positive impact on Behavioral Intentions.	0.215	8.356	0.000***	Supported
H3: Social Influence has a positive impact on Behavioral Intentions.	0.389	8.242	0.000***	Supported
H4: Facilitating Conditions have a positive impact on Behavioral Intentions.	0.192	14.523	0.000***	Supported
H5: Hedonic Motivation has a positive impact on Behavioral Intentions.	0.101	2.770	0.006***	Supported
H6: Price Value has a positive impact on Behavioral Intentions.	0.117	1.135	0.256	Not Supported
H7: Habits have a positive impact on Behavioral Intentions.	0.299	0.679	0.497	Not Supported
H8: Trust has a positive impact on Behavioral Intentions.	0.147	2.292	0.022**	Supported
H9: Perceived Privacy has a positive impact on Behavioral Intentions.	0.218	2.944	0.003***	Supported
H10: Perceived Security has a positive impact on Behavioral Intentions.	0.353	1.090	0.276	Not Supported
H11: Perceived Intelligence has a positive impact on Behavioral Intentions.	0.429	7.322	0.000***	Supported
H12: Perceived Anthropomorphism has a positive impact on Behavioral Intentions.	0.516	7.541	0.000***	Supported
H13: Behavioral Intentions have a positive impact on Use Behavior.	0.368	12.345	0.000***	Supported

***/**/* specify significance at 99 percent, 95 percent, and 90 percent confidence levels.

## 5 Discussion and implications

### 5.1 Discussion of the hypothesis results

The results of the structural model study provide insight into a number of critical variables that affect users’ behavioral intents to utilize mobile banking.

Performance expectancy (H1), or the degree to which users believe that using mobile banking will enhance their banking efficiency, was found to significantly impact behavioral intentions. This result aligns with existing literature, such as the studies by Abu-Taieh et al. (2022), Kumar et al. (2023), and Martins et al. (2014), and confirm that performance expectancy is a strong predictor of technology adoption. In the context of mobile banking, users are more likely to adopt the technology if they perceive it will improve the speed and effectiveness of their banking tasks. This finding extends the literature by emphasizing how performance expectancy specifically influences mobile banking adoption, and provides insights on enhancing user experience. Recognizing the importance of performance expectancy is vital for providers as it can lead to higher adoption rates and greater financial inclusion.

Effort expectancy (H2), or the ease of using mobile banking applications, also showed a positive effect on behavioral intentions. This finding aligns with research by Al Tarawneh et al. (2023), Abu-Taieh et al. (2022), and Bhatiasevi (2016), which demonstrated that the simplicity and user-friendliness of mobile banking apps are critical for user acceptance. Users are more inclined to adopt mobile banking if the interface is intuitive and the transaction process is straightforward. This result extends the literature by emphasizing the significant role that effort expectancy plays in the specific context of mobile banking. It highlights that even small improvements in usability can lead to higher adoption rates. Moreover, in the post-COVID era, where digital banking has become essential, this understanding is even more important. Ensuring that mobile banking applications are user-friendly not only enhances customer satisfaction but also fosters greater financial inclusion. This focus on effort expectancy can inform design practices and policy decisions, which ensures that mobile banking solutions cater to a broad range of consumers. This results in higher engagement and usage.

Social influence (H3) significantly impacts behavioral intentions, which emphasizes the role of peer recommendations and norms in mobile banking adoption. This result supports findings by Al Tarawneh et al. (2023), and Baptista and Oliveira (2015), which highlighted that social influence, including opinions from family and friends, can significantly shape individuals’ decisions to use m-banking services. To improve user experience, m-banking developers might prioritize making their platforms more functional and user-friendly. Furthermore, highlighting the benefits of mobile banking, such as its efficiency and convenience, can raise users’ perceptions of its advantages and promote adoption. In the post-COVID era, where many users are looking for reliable and efficient banking solutions, leveraging social influence can be a powerful strategy. Encouraging satisfied users to share their positive experiences can create an effect, which enhances trust and increases adoption rates among potential users.

Facilitating conditions (H4) are strongly associated with behavioral intentions. This result highlights the importance of having the necessary resources and support systems for using mobile banking. This finding is consistent with studies by Al Tarawneh et al. (2023), and Nguyen et al. (2023), which showed that access to smartphones, internet connectivity, and adequate support are essential for the successful adoption of mobile banking. In the post-COVID era, where many consumers have turned to digital banking solutions, ensuring that users have the necessary support can significantly enhance their experience and confidence in using m-banking. By improving these facilitating conditions, banks can create a more inclusive environment, and encourage wider adoption.

Hedonic motivation (H5), or the enjoyment derived from using mobile banking applications, was found to positively influence behavioral intentions. This result is in line with research by Al Tarawneh et al. (2023), Andalib Touchaei and Hazarina Hashim (2024), and Baptista and Oliveira (2017), which indicated that the fun and pleasure associated with using mobile banking can enhance user adoption. Users are more likely to engage with mobile banking if they find the experience enjoyable and satisfying. This finding extends the literature by highlighting the significance of hedonic factors in the context of mobile banking. While previous research has recognized the role of enjoyment in technology adoption, this study underscores that hedonic motivation is particularly important in financial services.

Price value (H6) did not show a significant impact on behavioral intentions. It implies that the cost–benefit analysis is not a primary driver for adopting m-banking. This result is consistent with previous studies. Price value does not significantly affect behavioral intention to use mobile applications (Hew et al., 2015). Price value was found to have no significant impact on the intention to use mobile banking (Al Tarawneh et al., 2023). This result contrasts with findings by Morosan and DeFranco (2016), and suggests that while financial considerations are important, they may not be the most critical factor for users deciding to use mobile banking services. This finding extends the literature by challenging the conventional belief that cost and value are the primary motivators for technology adoption in financial services. It suggests that other factors, such as usability, social influence, and hedonic motivation, may outweigh financial considerations in the decision-making process for mobile banking (Andalib Touchaei and Hazarina Hashim, 2024). Additionally, m-banking applications are even free of charge by most banks (Al Tarawneh et al., 2023). This insight is particularly relevant in a post-COVID era, where users may prioritize convenience, accessibility, and the overall experience of using digital banking solutions over the costs associated with them. Culturally, Thai consumers often prioritize convenience, trust, and service quality over cost when adopting new technologies, especially in financial services where reliability is important. Additionally, the COVID-19 pandemic accelerated digital adoption out of necessity. It reduced users’ sensitivity to price considerations as they sought safe and accessible alternatives to traditional banking amid lockdowns and social distancing. Many users may have perceived mobile banking not just as a cost-saving option but as an essential tool for continuity and safety, diminishing the relative importance of price value in their adoption decisions.

Habits (H7) were not found to be significant, which indicates that past behavior might not strongly influence current intentions in the context of mobile banking. This finding contradicts studies by Oliveira et al. (2014) and Al Tarawneh et al. (2023), which showed that habitual behavior could impact technology adoption. Furthermore, mobile banking actual usage is significantly impacted by habit (Chetioui et al., 2023). The difference could be due to the rapidly changing nature of technology use post-COVID-19, where new habits are still forming. This result extends the literature by highlighting the potential volatility of user behavior in a digital world that has been significantly transformed by the pandemic. While traditional research emphasized the role of habits in guiding technology adoption, this finding suggests that in the post-COVID era, users may be more open to experimenting with new technologies rather than relying on past behaviors (Li et al., 2022). This shift indicates that mobile banking adoption is influenced more by current needs and preferences than by established habits. In Thailand, cultural tendencies emphasize adaptability and openness to new experiences, especially among younger populations, which could reduce reliance on past habitual behaviors when adopting new technologies. Moreover, the COVID-19 pandemic disrupted daily routines and forced rapid shifts toward digital tools, effectively breaking old habits and encouraging more deliberate decision-making about technology use. This disruption likely weakened the predictive power of habits, as users re-evaluated their behaviors in light of new necessities and environmental constraints.

Trust (H8) has a positive impact on behavioral intentions. This result underscores the necessity of trust in the security and reliability of mobile banking services for user adoption. This finding is consistent with research by Abu-Taieh et al. (2022), Kumar et al. (2023), and Yuen et al. (2015), which emphasized that trust is a critical factor that influences the acceptance and continued use of mobile banking. The importance of building trustworthiness in m-banking platforms is evident, as trust emerges as a strong predictor of behavioral intentions. This result confirms the literature by pointing out that in the rapidly evolving landscape of digital banking, trust may play a crucial role. As users increasingly rely on mobile banking for their financial needs, concerns about data privacy and security become central. The ongoing threats to cybersecurity and data breaches increase the need for financial institutions to establish robust trust frameworks that address user anxieties.

Perceived privacy (H9) significantly affects behavioral intentions, which reflects users’ concerns over data privacy and its importance in their decision to use mobile banking. A study by Hanif and Lallie (2021) support this finding, which highlights that users are more likely to adopt mobile banking if they believe their personal information is protected. This result emphasizes the critical role that perceived privacy plays in the adoption of mobile banking services. While existing studies have acknowledged the significance of privacy, this research highlights that users’ perceptions of their data protection directly influence their willingness to engage with mobile banking platforms. As digital financial services become increasingly prevalent, the importance of perceived privacy has only grown, particularly due to the rising public concern over data privacy.

Perceived security (H10) was not significant, which suggests that while security is a concern, it may not be a decisive factor for all users in the adoption of mobile banking. This result contrasts with findings by Almaiah et al. (2023), Hanif and Lallie (2021), Widyanto et al. (2022). Perceived security might be important, but not an individual determinant of behavioral intentions. This finding highlights the complexity of user motivations in mobile banking adoption. While previous studies have consistently emphasized the importance of security as a critical factor, this research suggests that users may weigh multiple influences, such as facilitating condition, social influence, and hedonic motivation, over perceived security when deciding whether to adopt mobile banking (Andalib Touchaei and Hazarina Hashim, 2024). This understanding reflects the dynamic user behavior in the post-COVID era, where other factors may play a more prominent role in shaping user intentions. In Thailand, many users trust established banks and their mobile apps. This trust may reduce worries about security risks. During the pandemic, people urgently needed digital services. Convenience and access became more important than security concerns. Users often assume that banks provide strong security by default. Also, increased digital literacy during COVID-19 made mobile banking feel normal and safe. These factors likely made security less important in deciding to use mobile banking.

Perceived intelligence (H11) significantly influenced behavioral intentions. It indicates that users value the intelligent features of AI in mobile banking services. This finding aligns with research by Huang and Rust (2018), which demonstrated that perceptions of AI’s capability to provide effective and personalized services enhance user acceptance. Perceived intelligence increases users’ willingness to adopt m-banking (Lee and Chen, 2022). The post-COVID-19 context is important as the pandemic has increased the necessity for efficient, contactless services, which pushes consumers toward digital banking solutions (Agarwal et al., 2024). During this period, consumers have developed a stronger appreciation for technologies that simplify their banking tasks and provide seamless experiences. AI’s perceived intelligence can ease concerns about managing finances remotely, which makes users more likely to engage with mobile banking apps. This trend underscores the importance for financial institutions to invest in AI capabilities that enhance perceived intelligence to ensure that their systems meet the expectations of post-pandemic consumers. This result highlights that perceived intelligence serves as a crucial driver in the adoption of mobile banking, especially in a rapidly evolving technological landscape. While previous studies have recognized the importance of user perceptions, this research emphasizes that AI’s capabilities to personalize and streamline banking experiences are important in attracting users.

Perceived anthropomorphism (H12) also had a significant positive impact. Hence, human-like interactions with AI in mobile banking can enhance user intentions to adopt these services. An article by Moussawi et al. (2021) supports this finding and shows that anthropomorphic AI can create more engaging and trustworthy user experiences. The pandemic has reshaped consumer interactions with technology, with a significant shift toward digital services (Cai et al., 2023). As face-to-face interactions declined, the need for technology that can replicate human-like engagement became more pronounced (Menon and Shilpa, 2023). Anthropomorphized AI in mobile banking can fill this gap by providing a more personalized and empathetic interaction, similar to speaking with a human bank representative. This human touch can help mitigate feelings of isolation and build stronger emotional connections with users. This can lead to greater trust and acceptance of mobile banking services in the post-pandemic world. This finding extends the literature by highlighting the role of anthropomorphism in technology acceptance, particularly in the financial sector. This study emphasizes that users are not just seeking efficiency, but they also desire relatable and emotional interactions. The research adds a new dimension to the UTAUT, and suggests that human-like characteristics in AI can enhance the usage of mobile banking.

Behavioral intentions (H13) were positively associated with actual use behavior. This result aligns with the recent literature on mobile banking adoption (Jadil et al., 2021; Kumar et al., 2024). It confirms that stronger intentions lead to a higher likelihood of using mobile banking. This result is consistent with the theory of planned behavior and other models of technology adoption, which posit that intentions are strong predictors of actual behavior. The positive association between behavioral intentions and actual use behavior supports the core premise of the UTAUT framework, which states that intention is a strong predictor of technology usage. This finding reinforces the idea that increasing favorable user attitudes, through improved user experience, trust, and AI integration, can translate into real engagement with mobile banking services. In the post-COVID-19 context, where digital adoption has accelerated, the intention-behavior link is especially relevant, as consumers increasingly rely on mobile platforms for financial transactions.

The COVID-19 pandemic has significantly altered the way consumers interact with technology and financial services. Trust, and privacy concerns have become more pronounced as users increasingly rely on digital platforms for essential transactions. At the same time, the pandemic accelerated the normalization of AI-driven services, which has influenced how users perceive and respond to intelligent systems. Features such as perceived intelligence and anthropomorphism now play a more central role in shaping behavioral intentions, as users seek not only functionality but also intuitive and human-like experiences. As a result, traditional predictors of technology adoption, like performance expectancy and effort expectancy, are now replaced by newer psychological and emotional dimensions influenced by the post-pandemic digital environment.

The COVID-19 pandemic has impacted the relationships between the studied factors and behavioral intentions in the model. Many relationships, such as those involving performance expectancy, effort expectancy, social influence, and facilitating conditions, were strengthened as lockdowns and social distancing measures forced individuals to rely more heavily on digital technologies for work, communication, and daily life. Conversely, factors like price value and habitual behaviors showed nonsignificant effects, likely because financial stress heightened price sensitivity but urgent needs for digital solutions dominated traditional cost considerations and disrupted established habits. The accelerated digital transition during the pandemic also heightened respondents’ awareness and acceptance of intelligent and anthropomorphic technologies, further reinforcing these factors’ influence. Security concerns appeared less influential, as users prioritized immediate access and convenience over potential risks. Respondents experienced considerable impacts from the pandemic, including prolonged lockdowns that limited physical interactions, economic hardships that affected spending behavior, and rapid shifts to online environments that increased exposure to and reliance on digital tools. These changes justify the study’s framing in the post-COVID-19 era to reflect a new behavioral landscape shaped by necessity, adaptation, and a reshaped relationship with technology adoption.

### 5.2 Implications for practice

This research is based on the UTAUT model and includes AI-related aspects. Users may be more willing to adopt mobile banking due to the integration of AI. AI technologies, such as machine learning and biometric authentication, significantly enhance security measures by detecting fraudulent activities in real time and providing secure authentication methods (Olabanji et al., 2024). This reassurance can alleviate users’ concerns about online banking security and encourage them to adopt mobile banking. Moreover, AI enables m-banking apps to offer personalized experiences tailored to individual users’ preferences and behaviors (Manser Payne et al., 2021). By analyzing user data, AI can provide customized financial advice, alerts about spending habits, and product recommendations. This level of personalization enhances user engagement and satisfaction, which makes users more inclined to adopt mobile banking as it feels more relevant and tailored to their needs. Furthermore, the convenience of conducting banking transactions anytime and anywhere is a significant driver for mobile banking adoption. AI enhances this aspect (Sheth et al., 2022) by automating routine tasks and enabling features like voice commands, which make banking even more accessible. Users are more likely to adopt m-banking if they perceive it as a more convenient option compared to traditional banking methods. The integration of AI into mobile banking enhances the effectiveness and precision of services, which can lead to better personalized experiences for users (Lin and Lee, 2023). AI algorithms can analyze large amounts of data quickly, and thereby allow mobile banking services to process transactions, identify fraud, and respond to customer inquiries in real time. This efficiency not only speeds up service delivery but also reduces errors. Thus, it ensures a more reliable banking experience. AI can tailor financial advice and product recommendations based on individual user behaviors, preferences, and goals. By analyzing spending patterns and financial habits, AI can provide insights that are specifically relevant to each user, which makes financial management more accessible and effective. AI technologies can be used by providers of mobile financial services to improve customer experiences, customize services, and accelerate workflows. Providers can increase user happiness and engagement by using AI-driven services like chatbots for customer service and predictive analytics for tailored suggestions (Madasamy and Aquilanz, 2023). The impact of AI’s perceived intelligence underscores the significance of AI-powered functionalities in improving the user experience on mobile banking platforms. Mobile banking companies may increase customer happiness and loyalty by using AI to create personalized solutions and anticipate user needs. Perceived anthropomorphism in AI has a strong positive effect. It indicates that users tend to attach human-like features to AI-powered interfaces, which increases trust and emotional involvement (Blut et al., 2021). Chatbots and virtual assistants that have been anthropomorphized by AI produce a feeling of familiarity and empathy that strengthens relationships with people. In order to improve user engagement and trust, mobile banking providers should take advantage of this phenomenon by creating AI interfaces that have human-like traits like empathy, humor, and conversational ability. M-banking companies can utilize AI to provide contextualized help, predictive insights, and personalized suggestions, which result in experiences that are suited to each user individually (Lee and Chen, 2022). M-banking providers may increase customer engagement, and loyalty by leveraging AI to create personalized, and reliable experiences. This could result in increased adoption and utilization of m-banking services in the post-COVID-19 world.

### 5.3 Implications for policymakers

Policymakers, including government agencies and financial regulators such as ministries and central banks, need to establish clear guidelines and regulations that govern the use of AI in financial services. These frameworks should address data privacy, algorithmic transparency, and ethical considerations to ensure fair and safe AI practices. Regulations, shaped by financial regulators and legislative bodies, should also mandate regular audits and assessments of AI systems to mitigate risks of bias and discrimination. Policies created by data protection authorities should prioritize consumer protection by ensuring that AI-driven decisions are explainable and fair. Financial institutions may provide clear disclosures about AI usage, data handling practices, and potential risks to empower consumers to make informed choices. Regulations should also mandate robust cybersecurity protocols to safeguard sensitive user information.

### 5.4 Implications for research

This research is grounded in the UTAUT model, which provides a robust framework for understanding the factors influencing user acceptance of technology. In this study, the constructs of the UTAUT are applied to the context of mobile banking. In addition to the traditional UTAUT constructs, this research integrates AI-related aspects to enrich the analysis further. As mobile banking increasingly incorporates artificial intelligence technologies, it becomes essential to understand how these innovations impact user perceptions and behaviors. Therefore, this study examines constructs such as perceived intelligence and perceived anthropomorphism. Previous models of UTAUT have primarily focused on constructs like performance expectancy, effort expectancy, and social influence to explain technology adoption. By integrating perceived intelligence and anthropomorphism, this study extends these models to better capture the impacts of AI-driven technologies. This integration provides a more comprehensive understanding of user acceptance in the context of modern, AI-enhanced digital services. Traditional acceptance models have largely emphasized functional attributes. The inclusion of anthropomorphism highlights the importance of psychological and emotional factors in technology adoption. It shows the necessity of considering these factors in future studies. Furthermore, integrating insights from psychology, human-computer interaction, and AI into information systems research can provide a richer understanding of user interactions with AI technologies. Research can guide financial institutions in developing innovative AI applications tailored to specific market segments and user needs.

### 5.5 Limitations and future research

The study’s emphasis on Thai respondents limits the results’ applicability to other cultural and environmental contexts. In order to identify particular elements that impact user behavior, future studies could examine the influences that affect the acceptance of m-banking in various geographic locations and cultural contexts. The sample was slightly skewed toward younger users. This overrepresentation of digital natives may have influenced the results. However, younger users are among the primary adopters and drivers of digital financial services in Thailand and globally, making them highly relevant to the research objective. This demographic is often the first to engage with AI-driven interfaces and digital platforms, which aligns well with the study’s focus on artificial intelligence constructs such as perceived intelligence and anthropomorphism. Moreover, the reliance on self-reported data introduces the risk of social desirability bias and subjective misreporting, although Harman’s single-factor test was conducted to assess common method bias. To mitigate these biases in future research, scholars could incorporate behavioral data (e.g., actual usage logs), or use indirect questioning techniques to reduce social desirability effects. This study used a cross-sectional design, capturing user behavior and intentions at a single point in time. A longitudinal approach would be useful for examining how behavioral patterns and influencing factors evolve. Future research could address these limitations to enhance the robustness and applicability of the findings. The study ignores any mediating and moderating variables that may have an impact on these connections in favor of concentrating on the direct relationships between predictors and behavioral intentions. Moreover, future studies could look into how consumers’ attitudes and actions about mobile banking are affected by new technologies like biometrics, blockchain, and augmented reality. Future research may also quantify AI differently and more thoroughly by adding new variables that are unique to AI. Researchers may, for example, look into how customers perceive AI by analyzing aspects like perceived responsiveness, dependability, and flexibility of AI-driven features in mobile banking platforms. Future research could explore in more detail why price value, habits, and perceived security showed no significant impact on behavioral intentions. Qualitative studies could provide better understandings into users’ perceptions of cost and security when adopting mobile banking. Expanding research to different regions or countries could reveal whether these null effects are specific to the Thai post-pandemic context or generalize more broadly. Further, future research could apply multi-group SEM to explore whether relationships differ across demographic groups such as age, education, or prior AI experience.

## 6 Conclusion

In conclusion, this study has provided valuable insights into the determinants of behavioral intentions to use m-banking. It employs structural equation modeling to analyze data gathered from 412 respondents in Thailand. Through the examination of various hypotheses, this research has contributed to a deeper understanding of the factors that impact users’ attitudes and behaviors in the context of m-banking adoption. The findings of this study highlight the significant positive effects of several key factors, including performance expectancy, social influence, effort expectancy, facilitating conditions, trust, perceived privacy, and perceived intelligence and anthropomorphism of AI, on users’ behavioral intentions to adopt m-banking. The results show how consumers’ behaviors are significantly impacted by perceived intelligence and the anthropomorphism of AI, which underscores the potential of AI technology in the mobile banking sector. Mobile banking companies may improve customer experiences, customize services, and accelerate processes with the help of artificial intelligence. Mobile banking providers can offer personalized solutions that connect with customers on a personal level and increase engagement and loyalty by utilizing artificial intelligence. The study also highlights how important emotional connection and trust are to AI-powered user interfaces. Feelings of familiarity, empathy, and trust are fostered by people attributing human-like characteristics to AI-driven features. By creating AI interfaces that resemble people, mobile banking companies may take advantage of this phenomenon and boost user engagement.

## Data Availability

The original contributions presented in the study are included in the article/Supplementary material, further inquiries can be directed to the corresponding author.
