# Supplementary figures and images for: The impact of artificial intelligence on behavioral intentions to use mobile banking in the post-COVID-19 era
Source: Front Artif Intell. 2025 Aug 12;8:1649392. doi: 10.3389/frai.2025.1649392 (PMC12378666; doi:10.3389/frai.2025.1649392)

**Appendix 1.** Questionnaires items


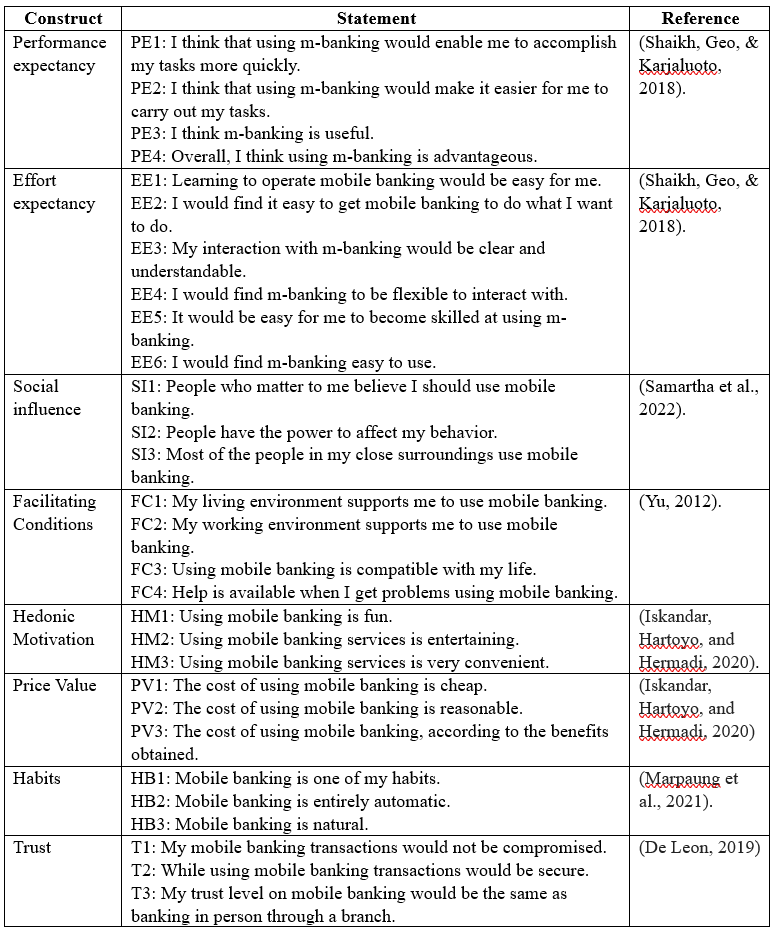


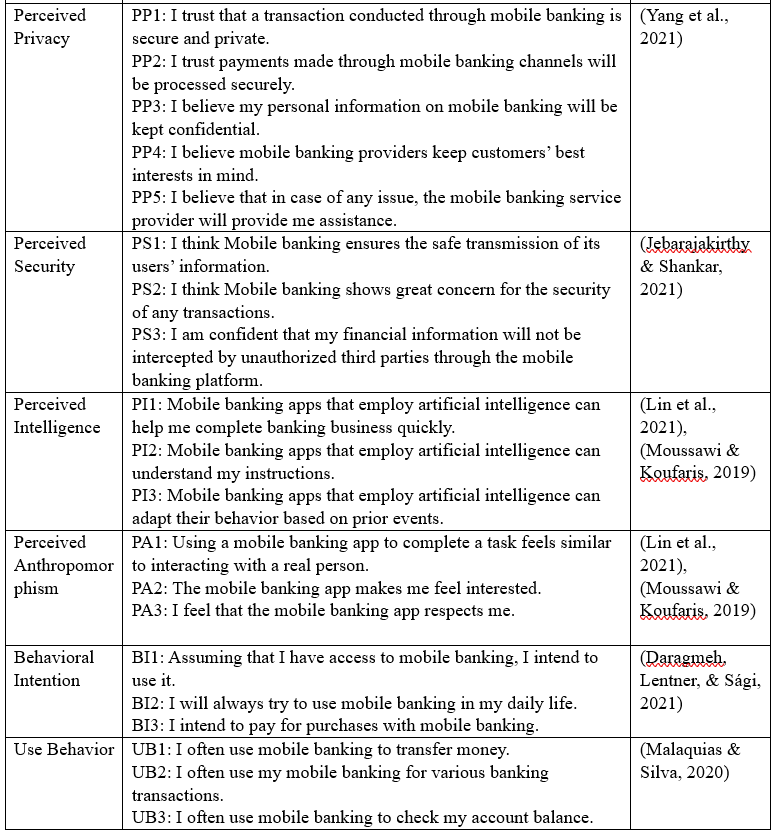

Supplement: Supplementary file 1 [file Table_1.docx]
